# Conference of the Southern Boards of Health

**Published:** 1898-11

**Authors:** 


### Conference of the Southern Boards of Health.

President Souchon of the Louisiana State Board of Health gives notice that he will call a meeting of the several Southern Boards of Health to be held in New Orleans, December 15, for the purpose of considering the best means to be adopted to prevent unwarranted quarantines between States or between counties or parishes.

It is extremely probable that we will have one or more health or quarantine conferences or conventions this winter,. The meeting called by Dr. Souchon for December is not, however, for the purpose of considering the subjects of national quarantine, the origin and character of the fever, etc. It is simply a conference between the health officers of the several States interested in the fever and affected by it to see if they cannot come to some agreement that will prevent unnnecessary and unjustifiable quarantine. It is very much like the Atlanta conference, which was similarly composed of the health officers of the Southern States, and which decided upon a plan of disinfection and a classification of freight as dangerous or not dangerous in epidemic years. The agreement reached at that conference was the only improvement made in quarantine rules and regulations last year, and it has operated most favorably. We are able to appreciate it today, for it has opened considerable trade that would otherwise be closed.

It is probable that by talking over the matter the several Southern Boards of Health can reach an agreement determining exactly when and under what conditions quarantine should be declared, and pledging themselves not to declare it unless these conditions exist. A great many hasty things are done in the midst of a panic. It is to prevent a repetition of these mistakes that Dr. Souchon calls the health officers of the States together. We do not think much difficulty will be encountered in bringing them to an agreement, and thus avoiding much of the hardship and injustice of this summer.—*Editorial in Times Democrat.*

There will also be held in Memphis, Tenn., during this month a quarantine convention for similar purposes. We hope that they may solve the difficult problem. The Atlanta method was given a trial by New Orleans this year "and it operated most favorably," says the *Times-Democrat,* and adds: "It opened considerable trade that would otherwise be closed." We may add, it also scattered infection, and at the time the editorial was written yellow fever was raging in some forty places all over two states. If anything has been more clearly demonstrated this fall than the inability of the Marine Hospital service to handle yellow fever, it has been that the Atlanta method will not work. The people can take their choice between trade and travel—and epidemic yellow fever. There has been no panic in Texas and no yellow fever. If yellow fever is a preventable disease, why have not the authorities of States, backed by the United States government, with a tremendous epidemic fund at disposal, prevented it? In the present state of knowledge non-intercourse seems to be the only preventive. We sincerely wish it were otherwise.—(Ed.)

